# Synthesis and Characterization of Exopolysaccharide Encapsulated PCL/Gelatin Skin Substitute for Full-Thickness Wound Regeneration

**DOI:** 10.3390/polym13060854

**Published:** 2021-03-10

**Authors:** Ahmad Hivechi, Peiman Brouki Milan, Khashayar Modabberi, Moein Amoupour, Kaveh Ebrahimzadeh, Amir Reza Gholipour, Faezeh Sedighi, Naser Amini, S. Hajir Bahrami, Alireza Rezapour, Masoud Hamidi, Cédric Delattre

**Affiliations:** 1 Department of Textile Engineering, School of Materials and Advanced Processing, Amirkabir University of Technology, Tehran 1591639675, Iran; hivehchi.a@iums.ac.ir (A.H.); hajirb@aut.ac.ir (S.H.B.); 2 Cellular and Molecular Research Center, Iran University of Medical Sciences, Tehran 1591639675, Iran; brouki.p@iums.ac.ir (P.B.M.); Amini.n@iums.ac.ir (N.A.); 3 Department of Tissue Engineering and Regenerative Medicine, Faculty of Advanced Technologies in Medicine, Iran University of Medical Sciences, Tehran 1591639675, Iran; 4 Department of Medical Biotechnology, Faculty of Paramedicine, Guilan University of Medical Sciences, Rasht 4477166595, Iran; khashayar.mdb@yahoo.com (K.M.); gholipoor.amirreza7@gmail.com (A.R.G.); faezeh.sedighi20@gmail.com (F.S.); 5 Department of Medical Biotechnology, Faculty of Allied Medicine, Iran University of Medical Sciences, Tehran 1591639675, Iran; moein.amoupour.ap@gmail.com; 6 Department of Neurosurgery, Loghman Hakim Hospital, Shahid Beheshti University of Medical Sciences, Tehran 1591639675, Iran; k.ebrahimzadeh@sbmu.ac.ir; 7 Skull Base Research Center, Loghman Hakim Hospital, Shahid Beheshti University of Medical Sciences, Tehran 1591639675, Iran; 8 Department of Tissue Engineering, School of Medicine, Qom University of Medical Sciences, Qom 3716993456, Iran; Alireza.rezapour@yahoo.com; 9 Université Clermont Auvergne, CNRS, Clermont Auvergne INP, Institut Pascal, F-63000 Clermont-Ferrand, France; 10 Institut Universitaire de France (IUF), 1 Rue Descartes, 75005 Paris, France

**Keywords:** nanofiber, wound dressing, exopolysaccharide, tissue regeneration

## Abstract

Loss of skin integrity can lead to serious problems and even death. In this study, for the first time, the effect of exopolysaccharide (EPS) produced by cold-adapted yeast *R. mucilaginosa* sp. GUMS16 on a full-thickness wound in rats was evaluated. The GUMS16 strain’s EPS was precipitated by adding cold ethanol and then lyophilized. Afterward, the EPS with polycaprolactone (PCL) and gelatin was fabricated into nanofibers with two single-needle and double-needle procedures. The rats’ full-thickness wounds were treated with nanofibers and Hematoxylin and eosin (H&E) and Masson’s Trichrome staining was done for studying the wound healing in rats. Obtained results from SEM, DLS, FTIR, and TGA showed that EPS has a carbohydrate chemical structure with an average diameter of 40 nm. Cell viability assessments showed that the 2% EPS loaded sample exhibits the highest cell activity. Moreover, in vivo implantation of nanofiber webs on the full-thickness wound on rat models displayed a faster healing rate when EPS was loaded into a nanofiber. These results suggest that the produced EPS can be used for skin tissue engineering applications.

## 1. Introduction

The skin, the largest organ in the body, it protects interior organs against toxins and environmental microorganisms and prevents dehydration in non-aquatic animals. Immune monitoring, sensory diagnosis, and self-treatment are other vital functions of the skin [1]. Skin accounts for about 8% of body weight, which varies in thickness from 1.5 mm to 4 mm and depends on the age of the individual and the area of the body. It can absorb, excrete, and is permeable to certain chemicals [2]. The skin is a complex organ with many essential functions; for instance, it acts as a mechanical barrier, participates in the body’s thermal regulation, helps initiate immunological processes, is involved in the production of melanin, and protects the body against UV radiation [2]. To accomplish all these functions, the skin has many different structures and contains diverse cells with different properties. The different skin layers include the epidermis, basal layer, squamous layer, granular layer, and cornified layer. Loss of skin integrity due to injury or illness can lead to severe physiological imbalances and, eventually, significant disability and even death [3]. The skin is highly susceptible to damage due to direct contact with the outside environment, so its immediate repair after injury plays a critical role in preventing future complications and pathogenicity [4].

It is estimated that annually about 35 million cases with significant skin losses require major therapeutic intervention. Of these, nearly 7 million have been diagnosed with chronic conditions. The most common cause of substantial skin loss is thermal injury, which is responsible for 300,000 deaths each year throughout the world. Other causes of skin loss include trauma, chronic ulcers caused by diabetes mellitus, pressure ulcers, and venous stasis [1,5]. Trauma to the skin can be divided into several degrees. The least trauma to the skin is to the epidermis layer, which is the most superficial layer of the skin; and the healing process is done by reshaping the epidermis, and there is no need for skin grafting. More severe trauma can result in partial or complete damage to the cutaneous and subcutaneous tissues [6]. Wounds that partially spread throughout the dermis are capable of healing, but unfortunately, the body cannot repair the deep skin damages adequately. In such cases, there are no cellular sources left for the skin to regenerate except in the area around the wound. Therefore, full wound healing takes a long time, leading to scar formation from the base of the wound [6]. The emergence of tissue-engineered skin alternatives has revolutionized the therapeutic potential for severe wounds and ulcers that do not want to be completely closed [7].

Tissue engineering is a field that aims to produce new biomaterials to repair damaged or diseased tissues or organs [8]. To achieve this, a cell source and experimentally produced extracellular matrix are required to support the cells. The tissue engineering method is cultured cells in vitro and placed on a porous scaffold that is eventually transplanted on the wound in vivo [9,10]. Synthetic or biologically composed dressings are often used to accelerate wound healing and improve the healing quality of chronic or burn wounds. Electrospinning has been known in the textile and fiber manufacturing industry for over 60 years [11,12,13]. In recent years there has been increasing interest in using this technology to produce nanoscale fibers for tissue engineering. Fibers have a porous structure that is suitable for drug delivery, gene delivery, and cell delivery [9,14]. Over the past decade, significant efforts have been made to develop nanofibers for tissue engineering that are made from biodegradable and biocompatible synthetic or natural polymers [15,16]. Ideally, a nanofiber candidate should be able to mimic the structure and biological function of natural extracellular matrix (ECM) proteins, thereby supporting and regulating cellular activity [17]. Also, nanofibers should determine and support the three-dimensional organization of tissue engineering space and maintain the normal state of differentiation within the cell compartment [17]. To achieve this, an engineered matrix must be biocompatible and should not have toxic effects on the surrounding tissue. Electrospun membrane skin replacements are the most advanced and efficient wound dressing material compared to modern bandages such as hydrocolloids, hydrogels, and alginates. These membranes have extraordinary properties such as controlled drug release and excellent cell growth because of their high specific surface area [18]. Many different biopolymers such as polysaccharides, bacterial polyesters, proteins, aliphatic polyesters, aliphatic polyamides, polydioxanone, polyorthoesters, polyanhydrides, and polyphosphazenes have been used in the electrospinning process to produce the aforementioned skin substitutes [17,19].

Polycaprolactone (PCL) has been used in a variety of tissue engineering applications. PCL is a biodegradable and biocompatible linear polyester that can be obtained either by ring-opening polymerization of ε-caprolactone or via ring-opening polymerization of 2-methylene-3-dioxepane [20,21,22]. Electrospun PCL has been known as an excellent candidate for skin substitute or wound dressing applications [23]. However, this polymer does not possess surface receptor sites for cell adhesion; therefore, it is often combined with other natural biopolymers [24]. Gelatin is a natural hydrophilic polymer and possesses natural cell adhesion sites because of its RGD sequences [25]. Thus a combination of these biopolymers will provide excellent mechanical and biological properties that are essential for full depth wound healing [25,26].

As mentioned earlier, polysaccharides have been used in the nanofiber scaffolds fabrication, which is called exopolysaccharides (EPSs) when excreted by an organism. EPSs can be divided into three groups according to their structural formula: (i) Homopolysaccharides: This type of polysaccharide comprises a constituent unit and can be divided into linear and branched types. An example of a linear homopolysaccharide is bacterial cellulose. Branched homopolysaccharides also include levans and dextrans; (ii) Heteropolysaccharides: This type of polysaccharides consist of one or more repetitive structural units with variable complexity, are well structured, and can also have short side chains; (iii) polysaccharides with abnormal structure: One of the best examples of this type of polysaccharide is alginate [27]. The EPSs produced by microorganisms are natural compounds that are widely used in the food, cosmetic, pharmaceutic, and chemical industries [28]. This material has been used as a thickener and emulsifier in the food and drug industries. In recent years, beta-glucans have been actively studied for their immunomodulatory, antitumor, antioxidant, and prebiotic activities [28]. All these functional properties exhibited by EPSs depend on their chemical structure, molecular weight, and concentration, which can be tuned using different microorganisms [29].

Polysaccharides are widely incorporated into nanofibers for different tissue engineering purposes. Hivechi et al. produced cellulose nanocrystal incorporated PCL/Gel hybrid nanofibers; their findings showed that this membrane influences the wound healing rate on the BALB/c mice incisional wound [30]. Rashtchian et al. also fabricated electrospun calcium alginate/PCL for wound healing application [31]. Baghersad et al., in a recent study, incorporated another polysaccharide source, aloe vera, into PCL/Gel nanofibers. In vivo investigation approved the positive effect of this polysaccharide on accelerated wound healing [32]. Ranjbar Mohammadi et al. also suggested gum tragacanth as a potential polysaccharide for drug delivery and wound regeneration process [33].

The GUMS16 EPS is a recently established and produced polysaccharide substrate which is not investigated thoroughly. To our knowledge, no research is published recently regarding the incorporation of the above-mentioned EPS. Therefore in this study, EPS was produced using the *R. mucilaginosa* sp. GUMS16 obtained from Deylaman forests [34]. Then it will be incorporated into PCL/Gel nanofibers using the electrospinning technique. Finally, the produced nanofiber membrane will be implanted in vivo on a full-thickness wound model for the first time. The healing rate and regeneration ability will be analyzed using histological techniques. We already know that produced EPS by the *R. mucilaginosa* sp. GUMS16 is a highly branched D-glucan that is composed of 85% glucose and 15% mannose units. Therefore, we hypothesize that fabricate EPS encapsulated PCL/Gelatin nanofibers will promote wound healing rate. This hypothesis will be evaluated using different in vitro and in vivo experiments.

## 2. Materials and Methods

### 2.1. Materials

Poly(caprolactone) (MW = 80,000 Da), potato dextrose broth (PDB, P6685), sucrose (98%, 252,603), ammonium sulfate (>99%, CAS#: 7783-20-2), phosphate-buffered saline (PBS, pH = 7.2–7.6, P4417) were obtained from Sigma Aldrich (Sigma Aldrich Chemical Co, Steinheim, Germany). Gelatin (CAS #: 9000-70-8), Acetic acid 100% (CAS #: 64-19-7), Hydrochloric acid (HCl 37%, 0.357 mol/L (1/2.8 N) Titripur^®^), ethanol 96%, and dimethyl sulfoxide (DMSO, CAS #: 67-68-5) were bought from Merck (Darmstadt, Germany). Other materials, such as Penicillin-streptomycin (10,000 U/mL, Gibco™ 15140122) and DMEM (Dulbecco’s Modified Eagle Medium, 15013LX) were purchased from Gibco (Gibco BRL Ltd, Paisley, Scotland).

### 2.2. Production and Isolation of EPS

Potato Dextrose Broth (PDB) culture medium was used for yeast culture and EPS production. This culture medium was prepared from a combination of PDB powder, sucrose, and ammonium sulfate in deionized water with concentrations of 24 g/L, 12.5 g/L, and 50 g/L, respectively. The pH of the resulting solution was then regulated at 6 by dropwise addition of 1 M HCL aqueous solution. The solution was autoclaved at 121 °C for 15 min at a pressure of 15 per square inch(PSI), and then they were stored at 4 °C. According to the procedure suggested by Hamidi et al. [28], the isolated *R. mucilaginosa* sp. GUMS16 was cultured on PDB plates and incubated for 24 h at 30 °C, followed by 72 h incubation at 4 °C. The resulting orange-colored single colonies on the culture plates were maintained in 30% glycerol for subsequent steps. The yeast was extracted from 30% glycerol (KalaZist Co., Tehran, Iran) stock and was added to Erlenmeyer flasks containing 100 mL of PDB media, and then transferred into a shaking incubator and incubated at 25 °C and a speed of 150 rpm. Then the culture media was transferred into two-liter Erlenmeyer flasks, and the volume of the culture medium was increased to 1 L with the addition of PDB solution. It was then incubated at 25 °C and 150 rpm for five days. The medium containing the cold-adapted yeast was centrifuged at 8500× *g* for 30 min at 4 °C. The supernatant was collected under sterile conditions. The EPS sedimentation process was carried out by slow addition of ethanol to the solution until the solution was four times diluted while it was agitating using a magnetic stirrer (IKA Werke GmbH, Staufen, Germany). The resulting mixture was then kept in the refrigerator at 4 °C for 24 h. The supernatant phase was then discarded, and the precipitate was transferred into falcon 50 mL conical centrifuge tubes containing ethanol and centrifuged at 8500× *g* and 4 °C for 20 min. The resulting precipitate was rewashed once again with cold ethanol and centrifuged at the above conditions. The resulting precipitate was then solubilized by adding deionized water and incubated overnight at −80 °C before being freeze-dried. Finally, it was freeze-dried at −60 °C and 5 mbar for 48 h.

### 2.3. Polymer Solution Preparation and Electrospinning

Acetic acid 90% (*v*/*v*) was used as the common solvent for both biopolymers. In samples containing EPS, the predetermined amount (0–2% on the weight of polymer) of this substance was first weighed and added to 5 mL of acetic acid 90%. Then the resultant mixture was sonicated at 68 kHz for 10 min using a Parsonic ultrasonic water bath (Pars Nahand, Iran) to obtain a stable suspension. Then, according to earlier reports [35,36], 0.85 g and 0.75 g of PCL and gelatin respectively were separately added to 5 mL acetic acid 90% and mixed for 5 h using a magnetic stirrer. PCL and Gel polymer solutions were mixed using a magnetic stirrer at a 3:2 volume ratio for an hour. The resultant solution was then transferred into a 5 mL syringe with a 22G needle and placed in a single nozzle electrospinning apparatus (Nanoazma, Tehran, Iran) equipped with a rotating cylindrical collector (Nanoazma, Tehran, Iran). Nanofibers were produced at 15 kV, 0.7 mL/h feed rate, 500 rpm collector speed, and 15 cm distance between the syringe tip and the collector.

### 2.4. Characterizations

#### 2.4.1. Morphological Studies

Surface characteristics of the fabricated nanofibers, such as morphology and diameter, were studied using scanning electron microscopy (SEM) images [37]. First, nanofibers were sliced into 5 × 5 mm^2^ squares using a sharp scissor. They were later mounted an SEM sample holder using double side adhesive carbon tape. The samples were coated by a thin layer of gold using an SC7620 sputter coater instrument (QUORUM TECHNOLOGIES LTD, Kent, UK). Images were taken at 20 kV accelerating voltage using a Hitachi S-4500 SEM apparatus. The diameter of the nanofibers was analyzed by ImageJ software, version 1.44.

#### 2.4.2. Fourier Transform Infrared (FT-IR) Spectroscopy 

Samples were mixed with potassium chloride (KBr) salt and milled into a fine powder using a ceramic porcelain pestle and mortar. A pellet of this powder was made under pressure and placed in a Thermo Nicolet (Nexus 670) FTIR apparatus (Thermo Nicolet, Madison, WI, USA). The spectrum of 40 scans was recorded in transmittance mode from 400–4000 cm^−1^ wavenumber.

#### 2.4.3. Dynamic Light Scattering

A 0.1 g EPS was added to 10 mL distilled water and sonicated for 15 min at 40 kHz. The hydrodynamic diameter and ξ-potential of the produced EPS were measured using a Malvern dynamic light scattering (DLS) apparatus (Malvern Instruments, Malvern, UK).

#### 2.4.4. Thermal Analysis

Thermal properties of the fabricated samples were investigated using a thermogravimetric/differential scanning calorimetry (TG/DSC) instrument (TA Instruments, Lukens Drive, NC, USA). Thermal properties were recorded from 25 °C to 700 °C at a 20 °C /min heating rate under inert gas purge (N_2_).

#### 2.4.5. Tensile Properties

A 40 × 5 mm^2^ rectangle was cut out of the nanofiber mesh and placed in an Instron 5566 (Instron Company, Norwood, MA, USA). Tests were performed at 5 mm/min crosshead speed, and five measurements were carried out for each sample.

#### 2.4.6. Water Contact Angle Measurement

Nanofiber webs were positioned on a flat surface, and a drop of water was poured on the surface by a micro-syringe. The contact angle was recorded 5 s after placing the water droplet using a PC conjugated Sony OCA15-plus camera (Sony, Tokyo, Japan). This experiment was repeated five times for each sample. Images were finally analyzed using ImageJ software.

#### 2.4.7. Cell Viability

To sterilize nanofiber webs, they were exposed to ultraviolet (UV) light for 2 h and then immersed in 70% ethanol for 30 min. Cell viability on these samples was quantitatively determined using 3-(4,5-dimethylthiazol-2-yl)-2,5-diphenyl tetrazolium bromide (MTT) assay. Webs were cut into 5 × 5 mm^2^ squares and placed in a 96-well cell culture plate followed by adding 200 µL of 8 × 10^5^ L929 fibroblast cells maintained in DMEM that was supplemented with 10% fetal bovine serum (FBS) and contained 100 U penicillin/streptomycin. Cell culture plates were incubated for 1, 2, and 3 days at 37 °C, 95% relative humidity, and 5% CO_2_. After each time interval, the old cell culture media was discarded, and 200 μL PBS solution containing MTT dye (0.5 mg/mL) was added to each well. Then they were incubated for one hour at 37 °C, 95% relative humidity, and 5% CO_2_ so that the MTT dye would react with the living cells and a purple precipitate would form. The PBS solution was removed and replaced with 200 μL dimethyl sulfoxide (DMSO) to dissolve the precipitated dye crystals. After removing the scaffold from each well, the absorbance at each well was recorded by Elisa Reader at 570 nm wavelength. The percentage of cell viability in the scaffold was determined by the following equation, Equation (1):

(1) $$\%Cell\;viability=\frac{N_{s}}{N_{c}}\;\times 100$$

where *N_s_* and *N_c_* are the number of live cells or, in other words, the absorbance in the scaffold and control sample, respectively. This experiment was replicated three times for each sample.

### 2.5. In Vivo Assessments

Animal trials were carried out under an IACUC approved research protocol at Iran University of Medical Science (IUMS) and were in accordance with the policy on Humane Care and Use of Laboratory Animals and Guide for the Care and Use of Laboratory Animals. In the first step, rats were anesthetized via intraperitoneal administration of ketamine Sigma Aldrich (St Louis, MO, USA) (91 mg/kg) and xylazine (Sigma Aldich, Steinheim, Germany) (9 mg/kg) combination. A sterilized disposable punch (6 mm in diameter) was used to create three full-thickness wounds between the two shoulders of each rat. The rats were divided into four groups. The first group of rats (group I) were treated with nanofibers without EPS and were regarded as the negative control. Group II and III were treated with nanofibers containing 1% and 2% EPS. Group IV rats were treated with Comfeel^®^ Plus (Coloplast, Humlebaek, Denmark) and were regarded as the positive control. Scaffolds were sutured to the wound area to prevent their detachment or replacement. All rates were covered with sterile gauze to prevent possible infection or scratching, and then they were kept in standard laboratory animal cages. Photographs of the wounded area were taken using a DSCW320 Sony^®^ digital camera (Sony, Tokyo, Japan) on 0.7, and 14 days to measure the wound closure. The photographs were taken by focusing the camera vertically to the middle of the wound, maintaining a 6 cm distance between the camera and wound. The wounded area and the contraction rate were then measured using ImageJ software. Rats were sacrificed by CO_2_ inhalation, and the wound area was harvested. Tissues were fixed using 10% neutral formalin buffer (Temad, Tehran, Iran) overnight. Then samples were dehydrated in ethanol, embedded in paraffin, and sectioned in 5 μm thickness using a Leica Biosystems microtome instrument (Leica Biosystems, Mount Waverley, Victoria, Australia). Finally, the prepared samples were stained with hematoxylin and eosin (H&E) and Masson’s trichrome according to the protocol suggested by Lee et al. Images of tissue sections were recorded via a PC coupled optical microscope.

### 2.6. Statistical Analysis

All data in this article are expressed as means ± standard deviation. SPSS software, version 16 was used for statistical analysis, and parameters with a *p*-value less than 0.05 were considered significant.

## 3. Results

### 3.1. EPS Characterization

**Figure 1:**
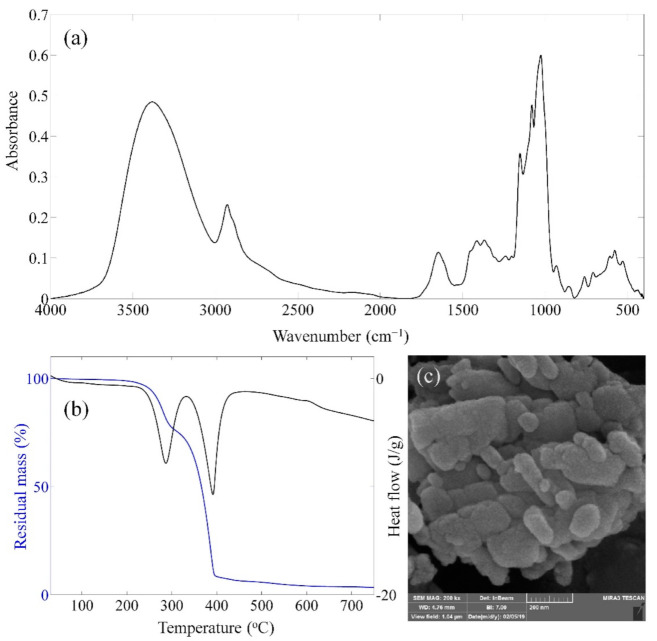
(**a**) FTIR spectrum, (**b**) TGA (blue)/DSC (black) diagram, and (**c**) SEM images of produced EPS.

Figure 1a demonstrates the FTIR spectrum of the produced EPS. According to Hamidi et al. [34], peaks at 3375 cm^−1^, 1647 cm^−1^, 1414 cm^−1^, and 1080 cm^−1^ are related to O-H stretch hydroxyl, C=O stretch, C-H bending (CH_2_), and S=O or C-O stretching functional groups. C-H stretch sp^3^, C-H bending (CH_3_), C-O stretch ether, and C-O stretch alcohol functional groups are noticeable at 2927 cm^−1^, 1369 cm^−1^, 1154 cm^−1^, and 1026 cm^−1^ vibrational peaks, respectively [31]. TGA diagram (Figure 1b) shows a two-step degradation process starting at around 280 °C and 350 °C with 20% and 65% weight losses, respectively. The final residual mass of EPS is about 3% indicating most of this substance degrades at 750 °C. DSC results showed two exothermic peaks at 280 °C and 390 °C. This finding suggests that EPS degrades in two steps releasing a significant amount of heat. SEM image of EPS particles is shown in Figure 1c. This image was processed using ImageJ software, and its results indicated that EPS particles exhibit an average diameter of around 40 nm. Hydrodynamic diameter of 42.7 nm was obtained from DLS results, which is in agreement with SEM findings. Moreover, the ξ-potential of the produced EPS was 4.83 mV, suggesting this material displays a positive surface charge.

### 3.2. Nanofiber Characterization

#### 3.2.1. Morphological Studies

**Figure 2:**
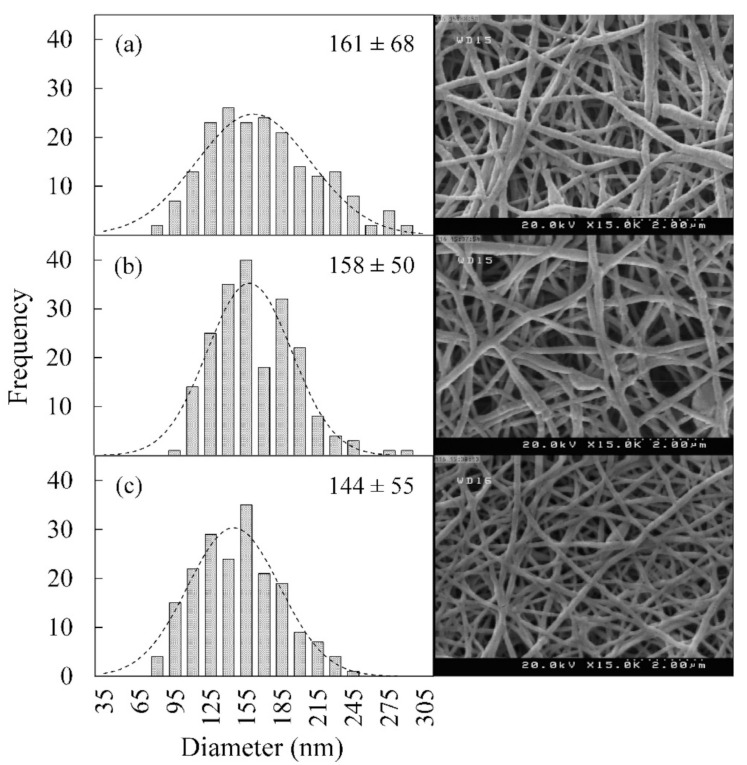
SEM images (**right**) and fiber diameter distribution (**left**) of the nanofiber samples (PCL/Gel) with different EPS contents: (**a**) 0% EPS (**b**) 1% EPS (**c**) 2% EPS.

Figure 2 demonstrates the morphology of the produced PCL/Gelatin (PCL/Gel) blend nanofibers encapsulated with 0–2% EPS. Bead-free smooth nanofibers were produced with a porous morphology, which is suitable for wound dressing application. Thus, contrary to Zhu et al. findings [38], the EPS incorporation does not reduce the electrospinability of the PCL/Gel blended system. The diameter of the nanofiber is a fundamental factor that directly impacts their properties, such as porosity, permeability, cell attachment, and degradation rate, which are essential for biomedical applications [39]. A standard Gaussian distribution curve was fitted on the frequency histogram. Results indicated that the EPS incorporation had significant effects on distribution curves. They became narrower (lesser standard deviation), and the mean nanofiber diameter dropped from 161 nm to 158 nm and 144 nm for samples containing 1% and 2% EPS, respectively. The nanofiber morphology can be affected by polymer solution properties, ambient conditions, and instrumental parameters [40]. In this article, all samples were prepared under the same environmental condition (25 °C, 65% relative humidity) and instrument parameters, the decrease in diameter could be attributed to changes in the polymer solution properties. Many researchers have investigated the effect of different polysaccharides incorporation on nanofiber morphology. Lin et al. [41] and Zhu et al. [38] reported that polymer solution viscosity decreases in Poly(ethylene oxide) (PEO) samples containing *dandelions* and *dendrobium officinale* carbohydrates, which resulted in thinner nanofibers. In contrast, Hivechi et al. observed that diameter increases in cellulose nanoparticle incorporated PCL [35] and gelatin [36] nanofibers because of increased viscosity. It is also reported that chitosan nanocrystals increase the nanofiber diameter [42], while starch nanoparticles showed the opposite trend [43]. To sum up, polysaccharide properties and their interaction with the blending polymer chains will affect solution properties, including viscosity, surface tension, and conductivity, which consequently changes the nanofiber morphology. The PCL/Gel polymer solution viscosity will rise, as it is expected when EPS solid particles are suspended [36]. Besides, bacterially produced polysaccharides are reported to possess negative zeta-potential [41]. Therefore, conductivity will increase with EPS incorporation resulting in stretched nanofiber during the electrospinning process. The conductivity overweight viscosity effect in our system and nanofibers became finer at higher EPS contents [38].

#### 3.2.2. FTIR Spectroscopy

**Figure 3:**
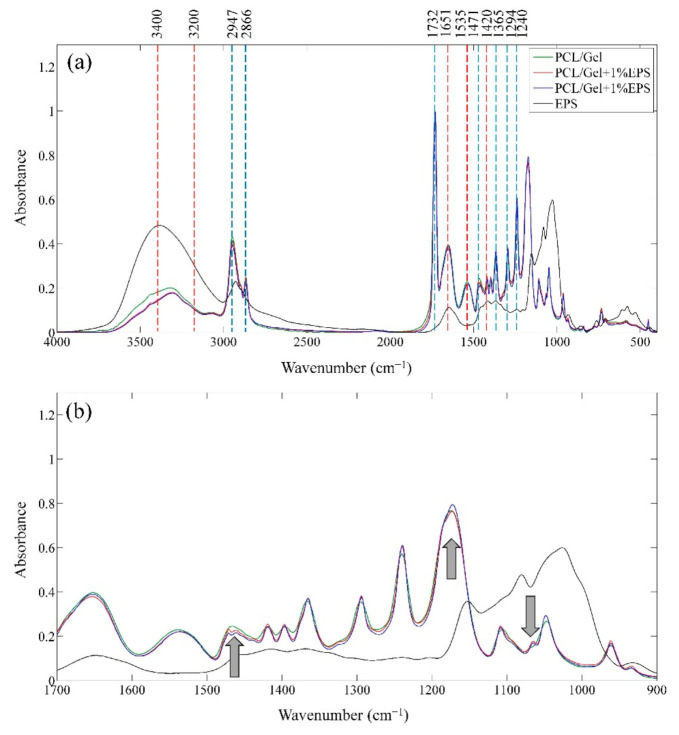
(**a**) Full (400–4000 cm^−1^) and (**b**) magnified spectrum (900–1700 cm^−1^) of EPS and PCL/Gel nanofibers containing different amounts of EPS. Blue and red dashed lines are the functional groups related to the PCL and gelatin portion of the nanofibers.

Chemical functional groups of the produced nanofibers were analyzed using FTIR spectroscopy, and its results are presented in Figure 3a. The spectrums related to PCL/Gel, PCL/Gel + 1% EPS, and PCL/Gel + 2% EPS are normalized to the peak at 1732 cm^−1^. The C-H stretch sp^3^, C=O stretch ester, C-H bending (CH_2_), C-H bending (CH_3_), C-C bonds in crystalline phase, and C-O-C stretch asymmetric-symmetric vibrational bonds of polycaprolactone (PCL) can be detected at 2866–2947 cm^−1^, 1732 cm^−1^, 1471 cm^−1^, 1365 cm^−1^, 1294 cm^−1^, 1240–1174 cm^−1^, respectively [44,45]. The observed peaks at 3200–3400 cm^−1^, 1651 cm^−1^, 1535 cm^−1^, and 1420 cm^−1^ are respectively representative of N-H stretch amid, C=O stretch amid, N-H bending, and C-N stretch amid of the gelatin [46]. The shape of the FTIR spectrum and its significant peaks were consistent with an earlier report by Hivechi et al. [30] that produce cellulose nanocrystal incorporated PCL/Gel hybrid nanofibers. Therefore, it can be concluded that blended PCL/Gel nanofibers are produced successfully.

Polysaccharide peaks are not detectable as separate peaks in the nanofibers spectrum due to the low amount of this substance in the system, as well as overlapping with PCL and gelatin FTIR peaks. The fingerprint region at 900–1700 cm^−1^, is magnified to achieve higher detection limits (Figure 3b). Three changes have been found in the fingerprint region (shown by arrows). The 1080 cm^−1^ peak of EPS can be seen as a shoulder in PCL/Gel-1% EPS and PCL/Gel-2% EPS, which was not present in the PCL/Gel sample. Besides, the peak 1174 cm^−1^ of PCL/Gel became weaker due to the low absorbance of EPS in this wavelength. Also, the shape of the single peak of the PCL/Gel at 1468 cm^−1^ is changed into a doublet with a maximum at 1470 cm^−1^ and 1460 cm^−1^ that can happen because of overlapped EPS peak. Therefore, it can be concluded that EPS encapsulated nanofibers have been produced successfully.

#### 3.2.3. Tensile Properties

Fabricating a tissue engineering scaffold with similar tensile properties carries the same weight as tailoring their biochemical characteristics since cells tend to grow on substrates with specific mechanical properties [47]. Table 1 demonstrates the summarized results obtained from five replicates of the stress-strain curve for different nanofiber samples. Analysis of variance (ANOVA) statistical studies revealed that the EPS incorporation did not change modulus and tensile strength (*p* > 0.05), while elongation at break is dropped significantly from around 28% to 14% (*p* < 0.05). Our finding was in contrast with Zhu et al. [38] study that reported increased elongation at break and tensile strength for polysaccharide incorporated PEO nanofibers. This inconsistency can happen due to the difference in polymer systems. Moreover, nanofibers diameter has decreased in this study, which logically will lead to lower elongation at break. Skin is relatively soft, and under uniaxial strain, shows a three-phase curve [47]. The Modulus, tensile strength, and elongation at break for skin range between 14–140 MPa [48], 2.5–16 MPa [49], and 20–70% [50], respectively. The mentioned values are suggesting that although nanofiber meshes became brittle after EPS addition, their mechanical properties are still acceptable as a wound dressing. This change in nanofiber’s mechanical properties could be attributed to the EPS agglomeration, resulting in weak points in the nanofiber membrane and reducing elongation at break.

#### 3.2.4. Water Contact Angle

**Figure 4:**
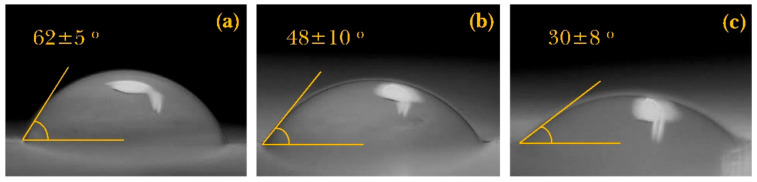
Water contact angle images (mean ± standard deviation) of (**a**) PCL/Gel, (**b**) PCL/Gel + 1% EPS, and (**c**) PCL/Gel + 2% EPS nanofiber web.

A suitable wound dressing should benefit from excellent hydrophilic properties so that it can absorb wound exudates and provide a proper surface environment for cell attachment [51]. Water contact angle measurement was used in this article to investigate the effect of EPS incorporation on surface hydrophilicity. Figure 4 demonstrates that the water contact angle decreased significantly with EPS incorporation. Since the nanofiber substrate for all samples is the same (PCL/Gel), it can be inferred that EPS has enhanced the water absorption capability. These results were in agreement with an earlier manuscript by Baghersad et al. [32,52], who reported a decreased contact angle after aloe-vera gel addition to the nanofibers. Many reports confirm enhanced water uptake properties by incorporating various types of polysaccharides [53]. This phenomenon can be attributed to the chemical structure of the polysaccharides and multiple hydrophilic functional groups such as hydroxyl, which causes more hydrogen bonds. As a result, more water will be absorbed into the nanofibers [54].

#### 3.2.5. Cell Viability

**Figure 5:**
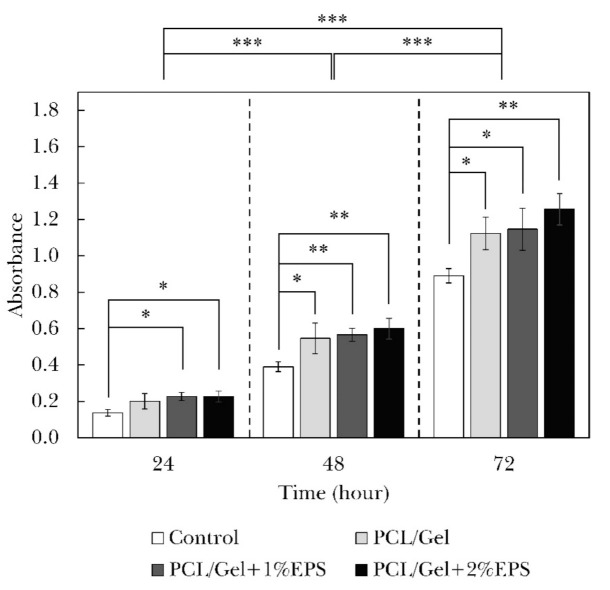
Results and statistical analysis of the cell viability examination obtained from MTT assay for PCL/Gel nanofibers containing different amounts of EPS (* *p* < 0.05, ** *p* < 0.01, *** *p* < 0.001).

The MTT assay results and their statistical analysis are illustrated in Figure 5. Results are indicating that cell activity has significantly increased in three successive days (*p* < 0.001). The PCL/Gel nanofiber containing 1% and 2% EPS showed significantly higher cell viability after 24 h of cell culture (*p* < 0.05). The viability increased in all cell-cultured samples after 48 and 72 h so that all nanofiber samples showed statistically higher bioactivity than the control sample (*p* < 0.05). These results point to the fact that produced nanofiber webs are not only biocompatible but also boost cell growth to some extent. This phenomenon was predictable since many researchers have reported that nanofibers can greatly enhance the cell growth process, such as their morphology, activity, proliferation, and differentiation [55]. The cell viability slightly increased with the EPS addition, so that the sample containing 2% of the polysaccharide substance showed the most significant difference with the control sample. We believe that the EPS incorporation enhances the cell attachment because of augmented surface hydrophilicity as well as improved cell-surface bonding between cell receptors and EPS functional groups. It is well known that cell attachment is an essential factor that ultimately regulates fundamental cellular processes, such as cell survival, proliferation, or migration. The cell death through apoptotic signaling will be declined when cells are attached and stabilized properly to the surface by integrin receptors. The importance of polysaccharides in enhancing cell viability and attachment was reported earlier by Freitas et al. [56], Rodríguez et al. [57], and Matsumoto et al. [58].

### 3.3. In Vivo Assessments

**Figure 6:**
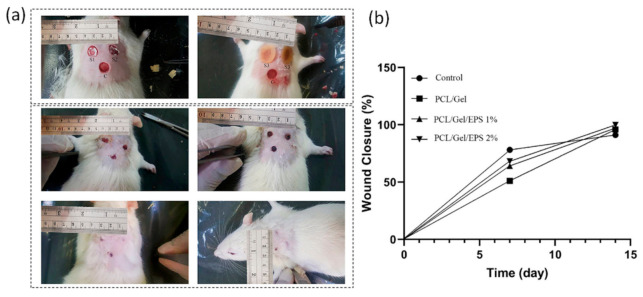
(**a**) Macroscopic images of wound area and closure rate (from 0 to 14 days) for the control and treated groups with PCL/Gel, PCL/Gel/1% EPS, and PCL/Gel/2% EPS nanofiber samples. (**b**) wound closure rate was measured 15 days post-surgery.

**Figure 7:**
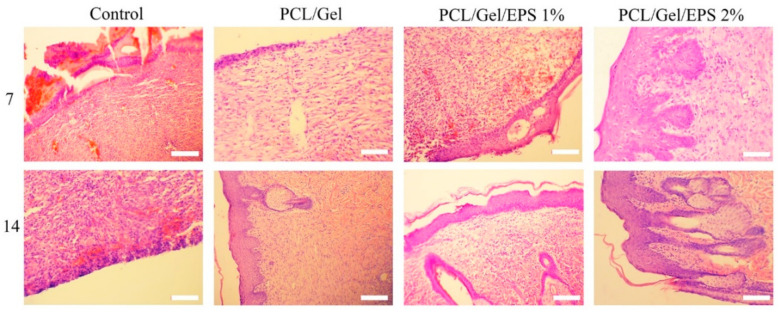
Microscopic images of Hematoxylin and Eosin-stained tissues after 7- and 14-days post-surgery.

The wound healing effects of nanofibrous scaffolds containing natural EPS are further examined in an incisional full-thickness skin defect model. Figure 6 demonstrates macroscopic images of wound area and closure rate of PCL/Gel, PCL/Gel/1% EPS and PCL/Gel/2% EPS groups. All treatment groups displayed wound closure to some extent, while PCL/Gel/2% EPS group showed the lowest wound area, which exhibited its healing effects on days 7 post-surgery. After two-week post-surgery, both PCL/Gel/1% EPS and PCL/Gel/2% EPS groups demonstrated better wound healing effect than PCL/Gel group. The healing rate of control (Comfeel^®^ Plus (Coloplast, Humlebaek, Denmark)), PCL/Gel, PCL/Gel/1% EPS and PCL/Gel/2% EPS, were 72.33 ± 2.1%, 82.1 ± 1.7%, 94.48 ± 2.3%, and 99.81 ± 1.39% respectively. Therefore, the results from EPS containing scaffolds exhibited their therapeutic effect than the control group by tracing the macroscopic evaluation via measuring wound closure rate, which was probably related to the synergic effects of the EPS, cell attachment and proliferation properties of nanofibers, and also the nanopatterning topography and microenvironment provided by fabricated wound dressing. EPS-incorporated wound dressings displayed the highest wound closure rate and lowest scar signs at the site of injury. The wound area in the PCL/Gel/1% EPS and PCL/Gel/2% EPS was rich in endothelial cells, fibroblast cells, and a newly formed epidermal layer could be observed (Figure 7). It is generally accepted that EPS has antioxidant properties, and it can scavenge ROS effectively that may diminish the inflammatory phase at the early phase of injury [59,60].

Wound healing progress was evaluated after staining of tissue sections with hematoxylin and eosin (H&E). As shown in Figure 7, mild infiltration of lymphocytes was seen in all groups on the seventh day post-surgery. PCL/Gel/1% EPS and PCL/Gel/2% EPS demonstrated relatively more granulation tissue with a high number of fibroblast cells and microvessels around the wound site, which is related to the anti-oxidant and anti-inflammatory effects of EPS. Complete re-epithelization was shown in all treated groups at days 14 post-surgery. Furthermore, EPS containing scaffold groups (PCL/Gel/1% EPS and PCL/Gel/2% EPS) exhibited more angiogenesis and dermal appendices, including hair follicles and sweat glands, than PCL/Gel and control groups. The granulation tissue and epithelial thickness were normal in both PCL/Gel/1% EPS and PCL/Gel/2% EPS when compared to other groups. Although there was no significant difference between PCL/Gel and the control group in vivo, PCL/Gel can act as a barrier for microbial infiltration into the wound bed result in suitable conditions and microenvironments for wound healing.

**Figure 8:**
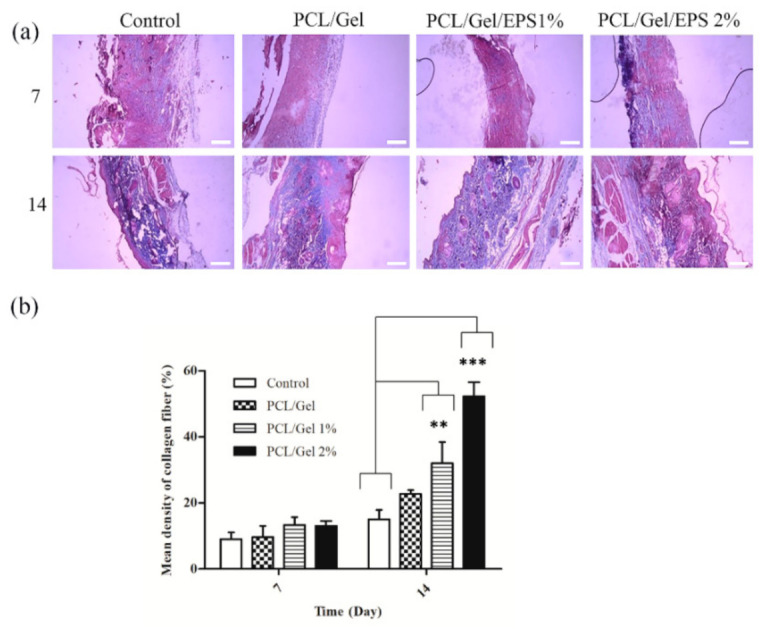
(**a**) Microscopic images of Trichrome Masson-stained tissues after 7- and 14-days post-surgery. (**b**) Statistical analysis according to the integrated optical density (IOD) value and mean density measured by Image-J software. Data are presented as the means ± standard deviation for at least three independent experiments. ** *p* < 0.01 vs. the control group, *** *p* < 0.01 vs. the control group.

The wound tissue of the control and treated groups were stained by Masson’s Trichrome stain method and the collagen fibers were stained blue (Figure 8). While most of the collagen in the control group was poorly organized and stained light blue with a disorganized arrangement, the EPS-treated groups exhibited to contain more organized (green) and they were stained dark blue stain with a highly ordered arrangement. As shown in Figure 8, the collagen content of PCL/Gel/1% EPS and PCL/Gel/2% EPS groups kept rising compared with other groups during the 14 days post-surgery. PCL/Gel/2% EPS group showed the highest collagen level among other groups. EPSs synthesized by microorganisms have previously been used to produce cosmetic creams due to their special functions such as emulsion stabilizers, skin conditioning materials, tensor, and antibacterial agent, anti-wrinkle and moisturizing properties [61,62]. In 2020, researchers [63] demonstrated that natural polysaccharides are isolated from a marine bacteria, *Pantoea* sp. YU16-S3, induced in vivo wound healing in a rat model through Wnt/β-catenin pathway and high expression of HB-EGF, FGF, E-cadherin, and β-catenin genes. An experimental demonstration of the antioxidant and antibacterial effects of EPS was also carried out by Trabelsi et al. [64] demonstrated that EPS produced by *Lactobacillus* sp. Ca6 led to wound regeneration in the excision wound model in rats. They found that a significant increase in hydroxyproline level and deposition and orientation of collagen fiber has occurred in the EPS treated groups when compared to the control group. Additionally, they reported the anti-inflammatory efficacy of EPS components in that study [65].

## 4. Conclusions

In this paper, EPSs isolated from *R. mucilaginosa* sp. GUMS16 was used for skin regeneration investigation. The produced EPS is a highly branched glucan that was characterized by FTIR, TGA, SEM, and DLS instruments. FTIR results confirmed the carbohydrate chemical structure of this product. Moreover, SEM and DLS findings suggested that EPS particles can be classified in nanomaterials with around 40 nm in diameter. EPS was then incorporated into PCL/Gel nanofiber to investigate their effect on the final properties of nanofiber scaffolds. The obtained results from SEM images showed that EPS incorporation decreases the average diameter of nanofibers. Moreover, the successful production of hybrid PCL/Gel chemical composition was confirmed using FTIR spectroscopy. Cell viability assessments indicated that 2% EPS incorporated nanofibers were significantly more viable for fibroblast cells. We believe cell attachment in this sample was increased because of its higher hydrophilicity. This factor is an essential factor that ultimately regulates fundamental cellular processes, such as cell survival, proliferation, or migration. Therefore, in EPS incorporated samples, cell viability increased due to higher hydrophilic properties. These samples were tested on rate models. Results of microscopic images showed that wounds treated with EPS incorporated samples showed a higher healing rate. H&E staining demonstrated relatively more granulation tissue with an increased number of fibroblast cells and microvessels around the wound site, which can be attributed to the antioxidant and anti-inflammatory effects of the EPS.

## Figures and Tables

**Table 1:** Tensile properties of PCL/Gel blend nanofiber containing 0–2% EPS.

Sample	Modulus (MPa)	Tensile Strength (MPa)	Elongation at Break (%)
PCL/Gel	135 ± 10	6.23 ± 0.20	28.2 ± 3.0
PCL/Gel + 1% EPS	116 ± 12	6.01 ± 0.69	19.6 ± 5.1
PCL/Gel + 2% EPS	137 ± 13	6.17 ± 0.20	14.0 ± 3.5

## Data Availability

Data sharing is not applicable to this article.
